# Supplementary material for: Crosstalk between Prostate Cancer Cells and Tumor-Associated Fibroblasts Enhances the Malignancy by Inhibiting the Tumor Suppressor PLZF
Source: Cancers (Basel). 2020 Apr 27;12(5):1083. doi: 10.3390/cancers12051083 (PMC7281005; doi:10.3390/cancers12051083)
Supplement: Supplementary file 1 [file cancers-12-01083-s001.pdf]

Supplementary Materials

Crosstalk Between Prostate Cancer Cells and Tumor-Associated Fibroblasts Enhances the Malignancy by Inhibiting the Tumor Suppressor PLZF

Kum Hee Noh, Ae Jin Jeong, Haeri Lee, Song-Hee Lee, Eun Hee Yi, Pahn-Shick Chang, Cheol Kwak and Sang-Kyu Ye

1. Supplementary Materials and Methods

1.1. Western Blot Analysis

Cell lysates were prepared using Triton X-100 lysis buffer (150nM NaCl, 20mM Tris, 1% Triton X-100, 1% sodium deoxycholate, 0.1% sodium dodecyl sulphate (SDS), 10mM ethylenediaminetetraacetic acid; EDTA) with protease inhibitors (NaF, Na_3VO_4, Leupeptin) and phenylmethanesulfonyl fluoride (PMSF). Lysates were separated via SDS-polyacrylamide gel electrophoresis and then transferred to nitrocellulose membranes (GE Healthcare, Pittsburgh, PA, USA). Membranes were incubated with blocking buffer (TBS with 5% skimmed milk and 0.1% tween 20) for 1h at room temperature followed by incubating at 4 °C overnight with tris-buffered saline and tween (TBST) and then incubated with secondary antibody in 1:10000 dilution (Enzo Life Science, Farmingdale, NY, USA) for 1h at room temperature. The signal was visualized using the ECL detection kit (SurModics, Eden Prairie, MN, USA). Primary antibodies against PLZF (Abcam, England), SHP-1 (Santa Cruz, CA), and phospho-STAT3(Tyr705), STAT3, phospho-JAK2(Tyr1007/1008), JAK2, phospho-TYK2 (Tyr1054/1055), TYK2 (Cell Signaling Technology, Danvers, MA, USA) were all diluted to 1:1000. GAPDH (Cell Signaling Technology, Danvers, MA, USA) antibody was used as loading control.

1.2. RNA Extraction, Reverse Transcription, and Quantitative Reverse Transcription Polymerase Chain Reaction (qRT-PCR) Analysis

Total RNA was isolated from cell line using RNAiso Plus reagent (Takara, Shiga, Japan) according to the manufacturer's instructions. cDNA was synthesized using the ReverTra Ace qPCR RT Master Mix (Toyobo, Osaka, Japan). The gene expression levels were detected by using EvaGreen qPCR Mastermix (Applied Biological Materials, Richmond, Canada) and performed real-time PCR on the Applied Biosystems 7300 Real-time PCR system (Life Technologies, USA) with the following PCR conditions; 95 °C for 2min, followed by 40 cycles of 95 °C for 15sec and 60 °C for 1min. Data were normalized to internal control GAPDH. Primers for PLZF (QT00029960), SHP-1 (QT00011725), STAT3 (QT00068754), E-cadherin (QT00080143), N-cadherin (QT00063196), Vimentin (QT00095794), Fibronectin (QT00038024), CCL3 (QT01008063), CCL4 (QT01008070), SerpinE1 (QT00062496), uPAR (QT00076447), MYC (QT00035406), CyclinD1 (QT00495285), BCL-2 (QT00025011), BCL-xL (QT00236712), MMP9 (QT00040040), MMP11 (QT00024031), PTPN2 (QT00020720) and GAPDH (QT01658692) were purchased from Qiagen (Germantown, MD, USA).

1.3. Plasmids, Small Interfering RNAs, and Transfection

The Empty vector or pcDNA3.1-PLZF plasmids were kindly given by prof. KC Jung (Seoul national university). Control vector or pcDNA3.1-PLZF vector were transfected into cells using Lipofectamine 2000 (Thermo Fisher Scientific, USA). Small interfering RNAs (siRNAs) were purchased from Qiagen (Germantown, MD, USA). siRNAs for PLZF (SI00083608, SI00083615, SI03090346), SHP-1 (SI04436831, SI04950407) were purchased from Qiagen (Germantown, MD, USA).

1.4. Cell Proliferation Assay

Cell Counting Kit (CCK) (Dongin-LS, Korea) was performed to detect the cell proliferation capacity. DU145 cells were plated at a density of 5 × 10^5 cells per well of a 6-well plate; LNCaP cells were plated at a density of 1 × 10^6 cells per well of a 6-well plate. For CCK assay, cells were given 24 h to attach and were transfected plasmid and siRNA 24, 48, 72 h. After transfection, cells were incubated with 100 µl/well of the CCK reagent for 2h at 37 °C and were measured the absorbance of the plates at 450 nm. All values were normalized to the vehicle control-treated wells.

1.5. Apoptosis and Cell Cycle Determination

For cell apoptosis assay, cells were stained with FITC Annexin-V and Propidium Iodide (PI) kit (Becton Dickinson Bioscience, San Jose, CA) according to the manufacturer's protocol. For cell cycle assay, cells were fixed in cold 70% ethanol and then stained with propidium iodide (Becton Dickinson Bioscience, San Jose, CA). Cells were then washed with PBS, resuspended in FACS buffer (Becton Dickinson Bioscience, San Jose, CA), and stained cells were analyzed using FACS LSRFortessa.

1.6. Wound Healing Assay

Cell migration was assessed by wound healing assay. In brief, cells (1 × 106 DU145 cells) in 6-well plates were allowed to reach confluence, and wounds were scratched using sterile tips. Wound closure was monitored every 24 h using a microscope and evaluated wound distances (100×, Nikon, Tokyo, Japan). The experiments were performed in triplication.

2. Supplementary Figures and Figure Legends

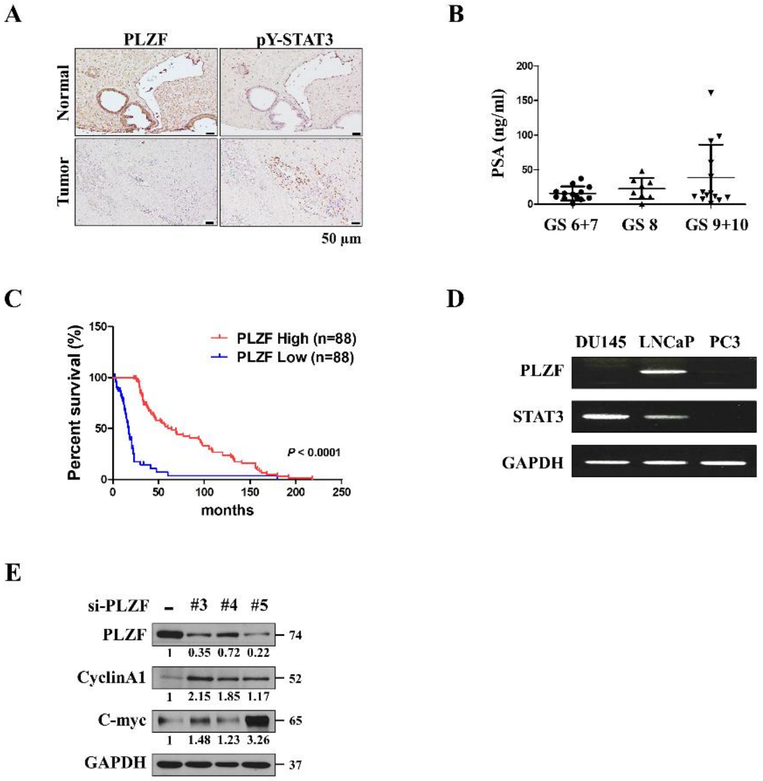

Figure S1. PLZF was downregulated in prostate tumor and negatively correlated with pY-STAT3. (A) Representative IHC staining image of normal prostate and prostate tumor tissues. (B) PSA levels were measured according to the Gleason scores. (C) Kaplan-Meier survival analysis of castrate-resistant prostate cancer (CRPC) patients according to PLZF expression. (D) PLZF, STAT3 and GAPDH mRNA expressions by RT-PCR in prostate cancer cell lines, DU145, LNCaP and PC3. GAPDH was used as a loading control. (E) LNCaP cells had been transfected with the siRNA at a 50nM concentration.

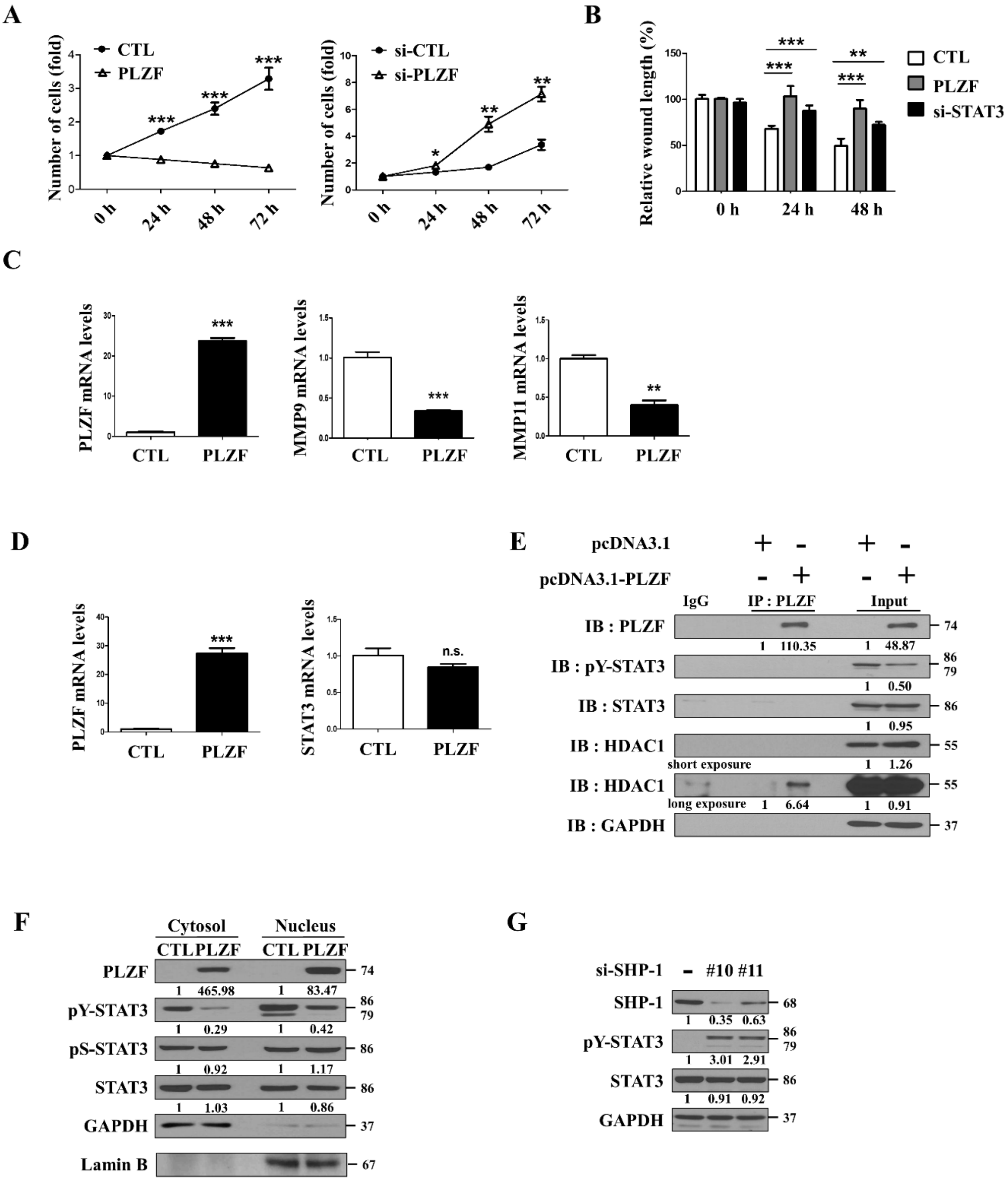

Figure S2. Exogenous transfection with PLZF reduces cell proliferation, migration ability and MMP9/11 which ECM component degradation enzymes. (A) Cell counting assay was conducted in PLZF plasmid and siRNA-transfected cells. (B) Wound healing assay was performed in PLZF plasmid and siSTAT3 transfected DU145 cells. The relative wound lengths are shown. (C) mRNA expression levels of PLZF, MMP9, MMP11 were examined by qRT-PCR in DU145 cells transfected with PLZF plasmid. (D) mRNA expression levels of PLZF, total STAT3 were examined by qRT-PCR in DU145 cells transfected with PLZF plasmid. (E) Detection of the interaction between PLZF and STAT3 (phosphorylation, total) by co-IP. The protein extract of PLZF was immunoprecipitated by anti-pY-STAT3 and anti-STAT3 antibody. The precipitate was subjected to Western blotting with the antibodies. HDAC1 is a positive control that is well known to form a transcriptional repressor complex with PLZF. (F) Representative Western blot image of the cytosol and nucleus expression in DU145 cells transfected with control-siRNA or PLZF plasmid for 48 h. (G) LNCaP cells had been transfected with the siRNA at a 50nM concentration.

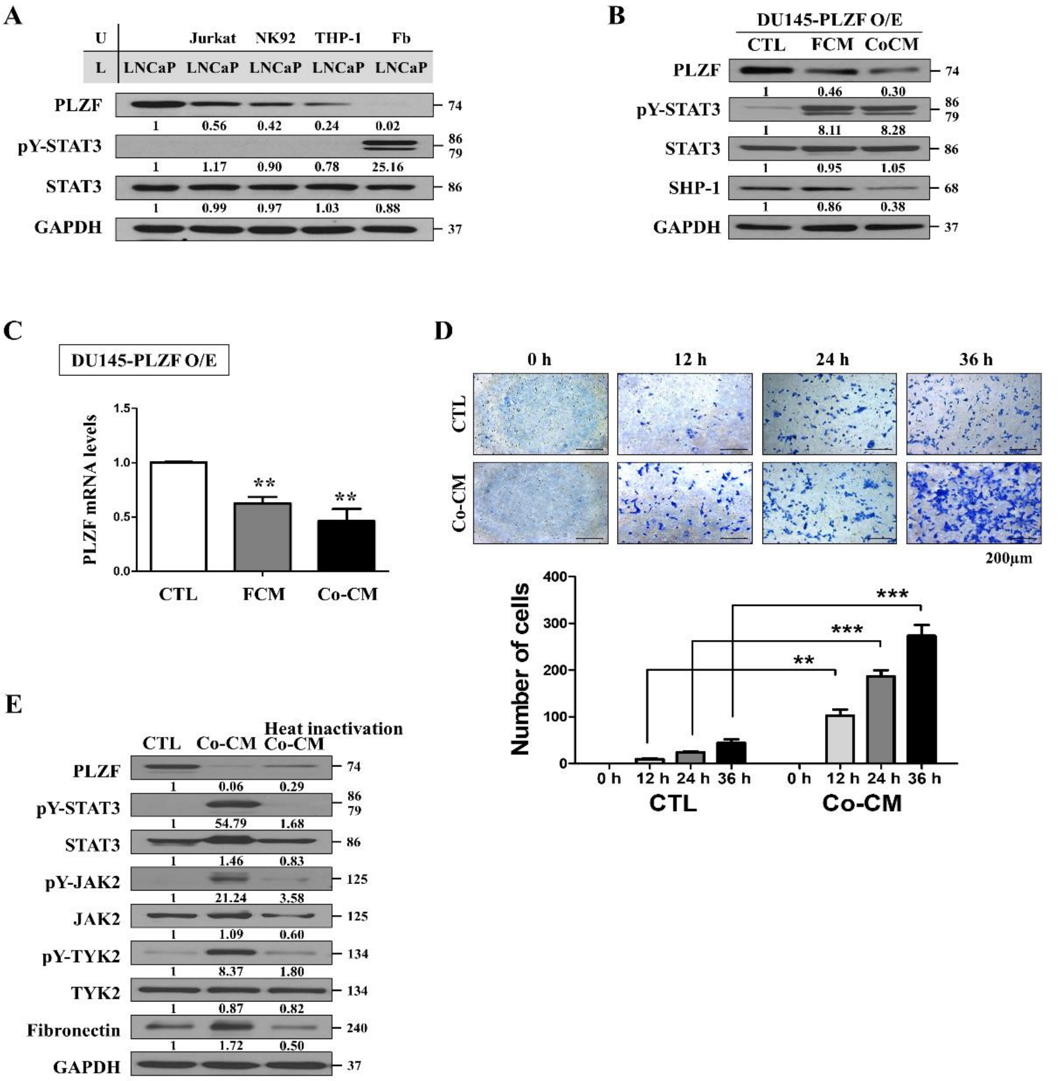

Figure S3. Prostate cancer cells are affected by fibroblasts to produce soluble factors and reduce PLZF expression. (A) LNCaP cells co-cultured with lymphocytes (Jurkat), NK cells (NK92), monocytes (THP-1) and fibroblast cells for 24 h. LNCaP cells were lysed for Western blotting. (B) PLZF-overexpressed DU145 cells with FCM or co-cultured with fibroblast for 24 h. DU145 cells were lysed for Western blotting. (C) mRNA expression levels of PLZF were examined by qRT-PCR in PLZF-overexpressed DU145 cells with FCM or co-culture. (D) Transwell matrigel invasion assays was conducted in LNCaP cells with Co-CM. The relative cell numbers are shown (bottom). Representative images from three independent experiments were quantified as mean ± SD. (E) Co-CMs were collected from LNCaP co-cultured with fibroblasts for 48 h. Heat inactivation Co-CMs were heated at 100 ℃ for 10min. Protein expression was subjected to western blotting.

A

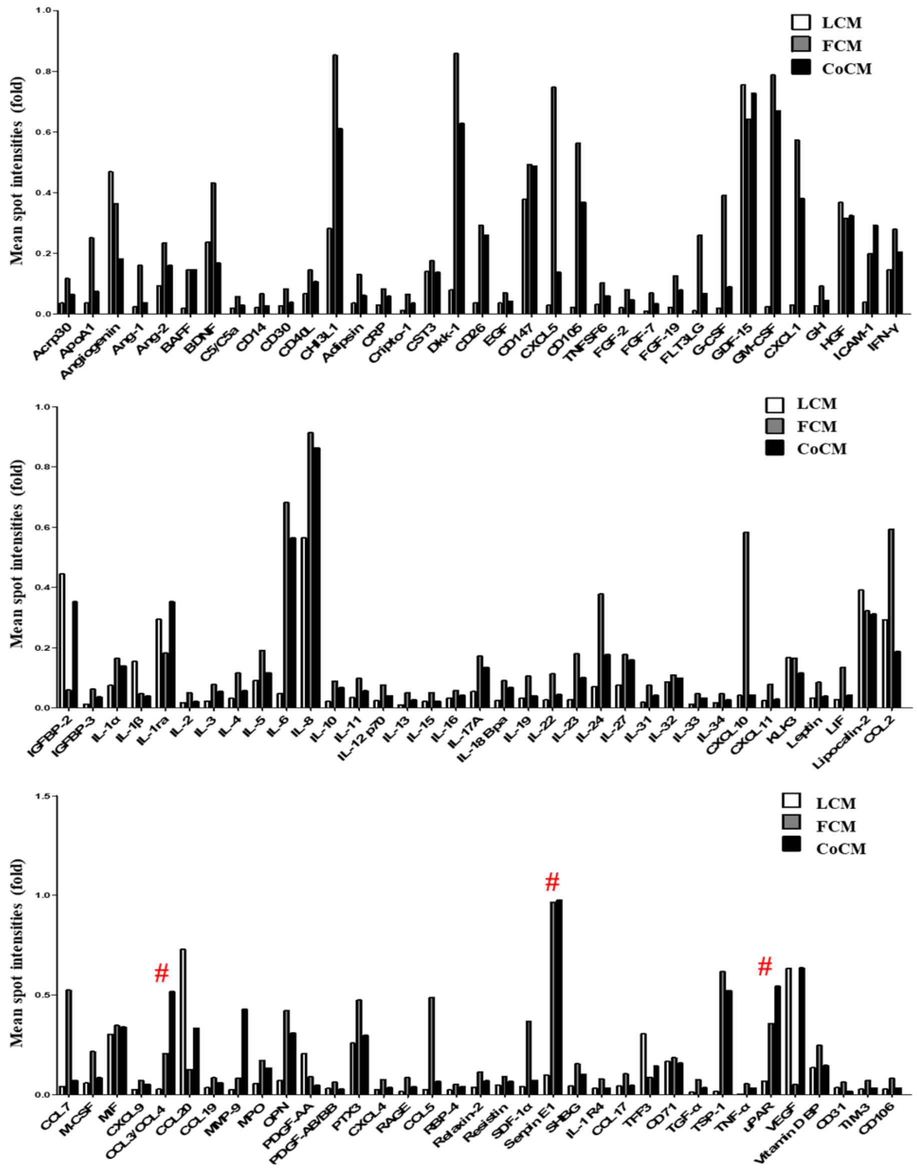
Mean spot intensities (fold)
LCM
FCM
CoCM
Acrp30
ApoA1
Angiogenin
Ang-1
Ang-2
BAFF
BDNF
C5/C5a
CD14
CD30
CD40L
CHI3L1
Adipsin
CRP
Cripto-1
CST3
Dkk-1
CD26
EGF
CD147
CXCL5
CD105
TNFSF6
FGF-2
FGF-7
FGF-19
FLT3LG
G-CSF
GDF-15
GM-CSF
CXCL1
GH
HGF
ICAM-1
IFN-γ
IGFBP-2
IGFBP-3
IL-1α
IL-1β
IL-1ra
IL-2
IL-3
IL-4
IL-5
IL-6
IL-8
IL-10
IL-11
IL-12 p70
IL-13
IL-15
IL-16
IL-17A
IL-18 Bpa
IL-19
IL-22
IL-23
IL-24
IL-27
IL-31
IL-32
IL-33
IL-34
CXCL10
CXCL11
KLK3
Leptin
LIF
Lipocalin-2
CCL2
CCL7
M-CSF
MIF
CXCL9
CCL3/CCL4
CCL20
CCL19
MMP-9
MPO
OPN
PDGF-AA
PDGF-AB/BB
PTX3
CXCL4
RAGE
CCL5
RBP-4
Relaxin-2
Resistin
SDF-1α
Serpin E1
SHBG
IL-1 R4
CCL17
TFF3
CD71
TGF-α
TSP-1
TNF-α
uPAR
VEGF
Vitamin D BP
CD31
TIM3
CD106
#
#
#

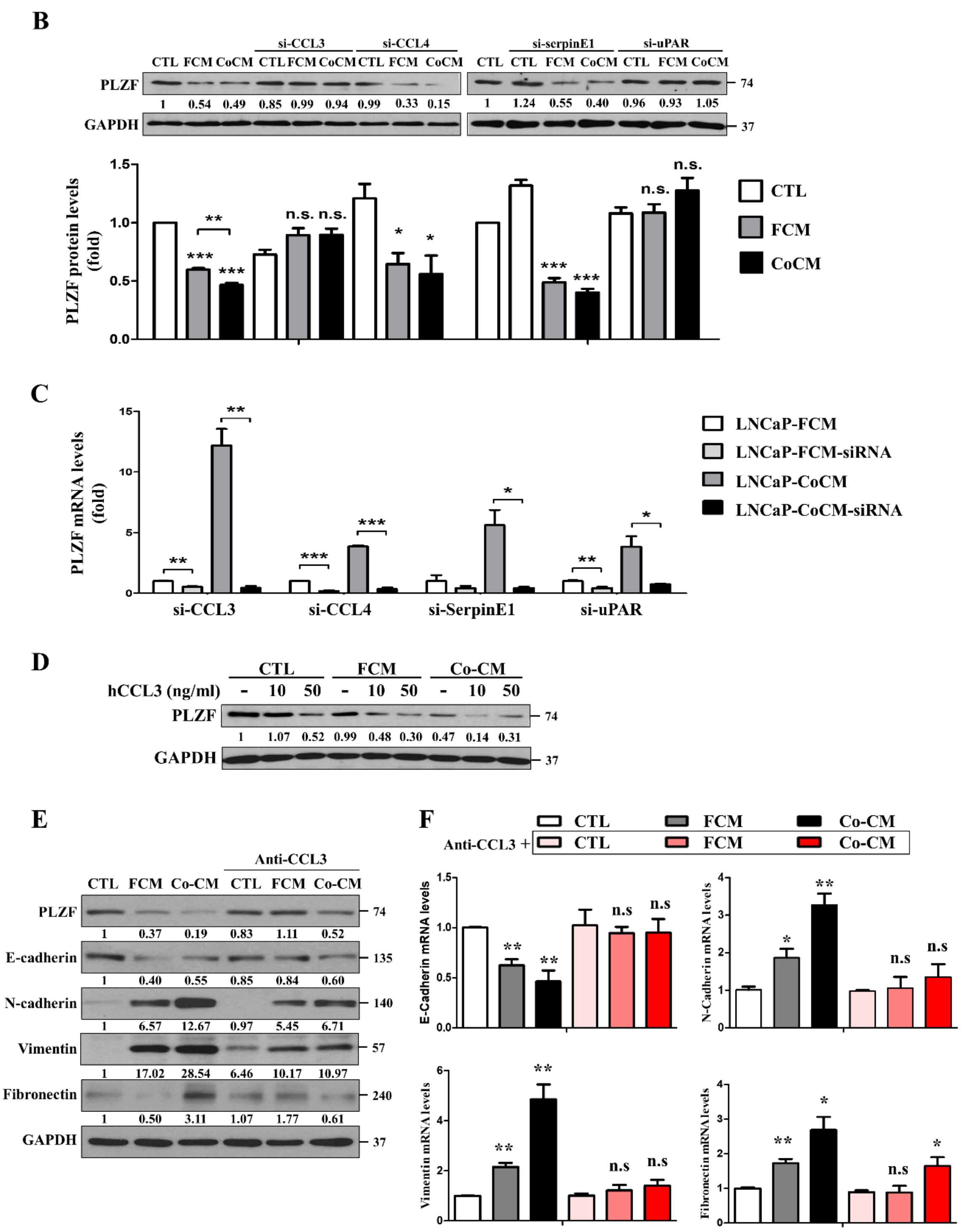

Figure S4. Identification of tumor associated fibroblast-derived cytokines. (A) The mean pixel density of each dot on ARY022B antibody array was quantified using the Adobe Photoshop. Mean intensities of cytokines are plotted. (B, C) CMs were collected from transfected fibroblast, LNCaP cells with 50nM siRNAs. LNCaP cells were cultured in the CMs for 24h and PLZF levels were identified to Western blotting (top) and qRT-PCR (bottom). (D) Cells were pre-treated with recombinant protein CCL3 for 4 h, incubated under FCM or Co-CM for 24 h. PLZF protein expressions were subjected to western blotting. (E) Protein expression levels of EMT markers in LNCaP cells with the indicated conditioned media with the addition of anti-CCL3 antibody. (F) mRNA expression levels of epithelial-mesenchymal-transition (EMT) markers were examined by qRT-PCR in LNCaP cells with the indicated conditioned media with the addition of anti-CCL3 antibody.

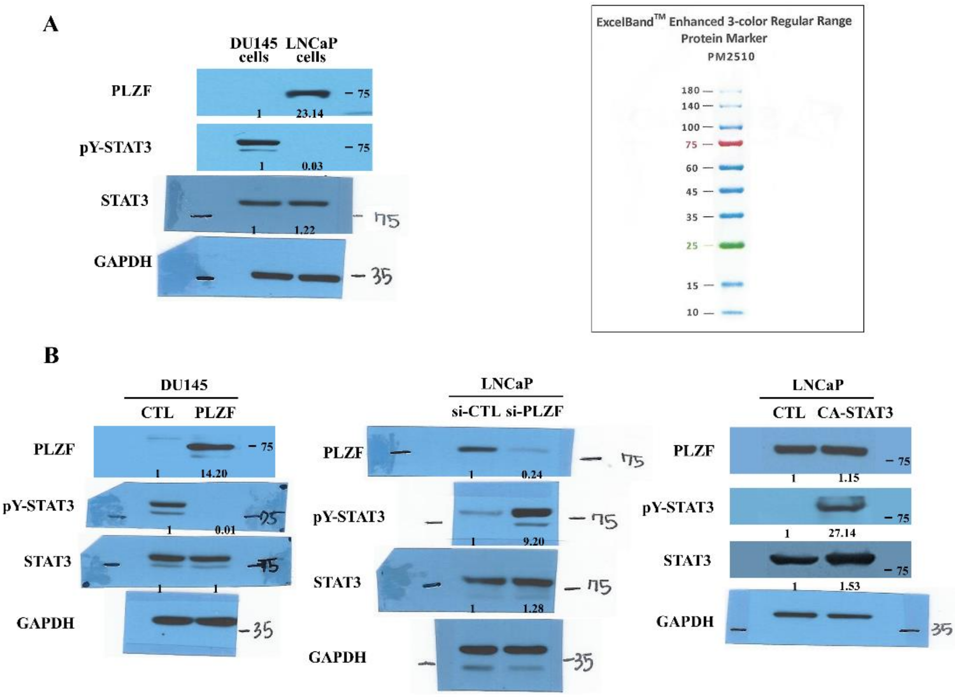

Figure S5. Original blots for western blot analyses shown in Figure 1: (A) Figure 1F, (B) Figure 1G.

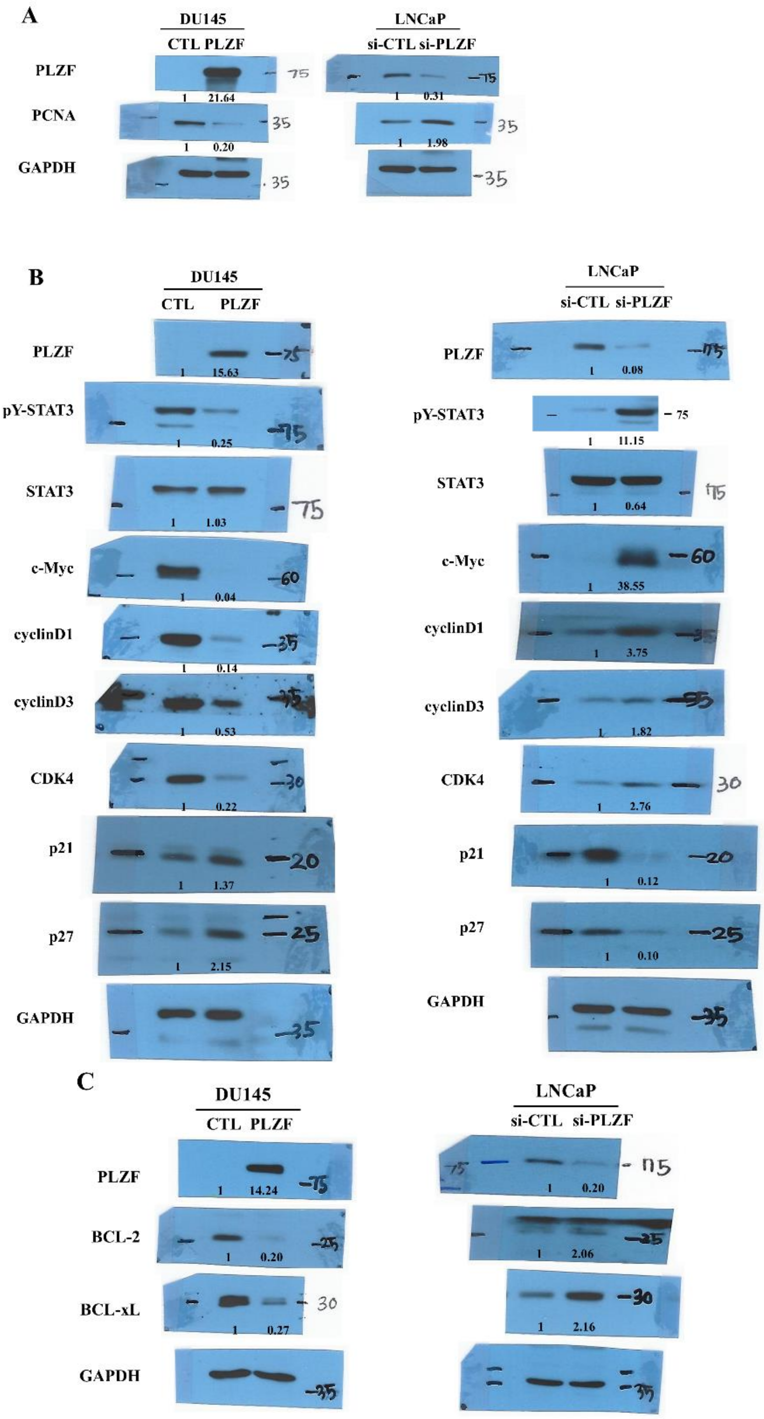

Figure S6. Original blots for western blot analyses shown in Figure 2: (A) Figure 2B, (B) Figure 2D, (C) Figure 2G.

A

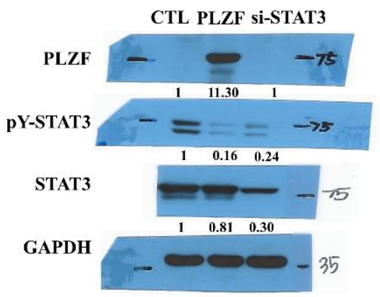

B

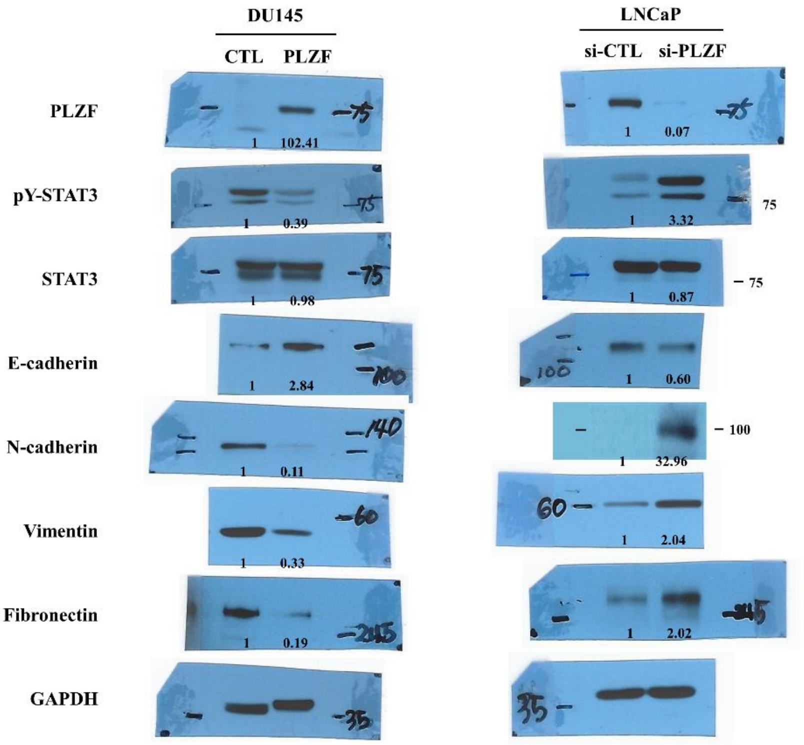

Figure S7. Original blots for western blot analyses shown in Figure 3: (A) Figure 3A, (B) Figure 3C.

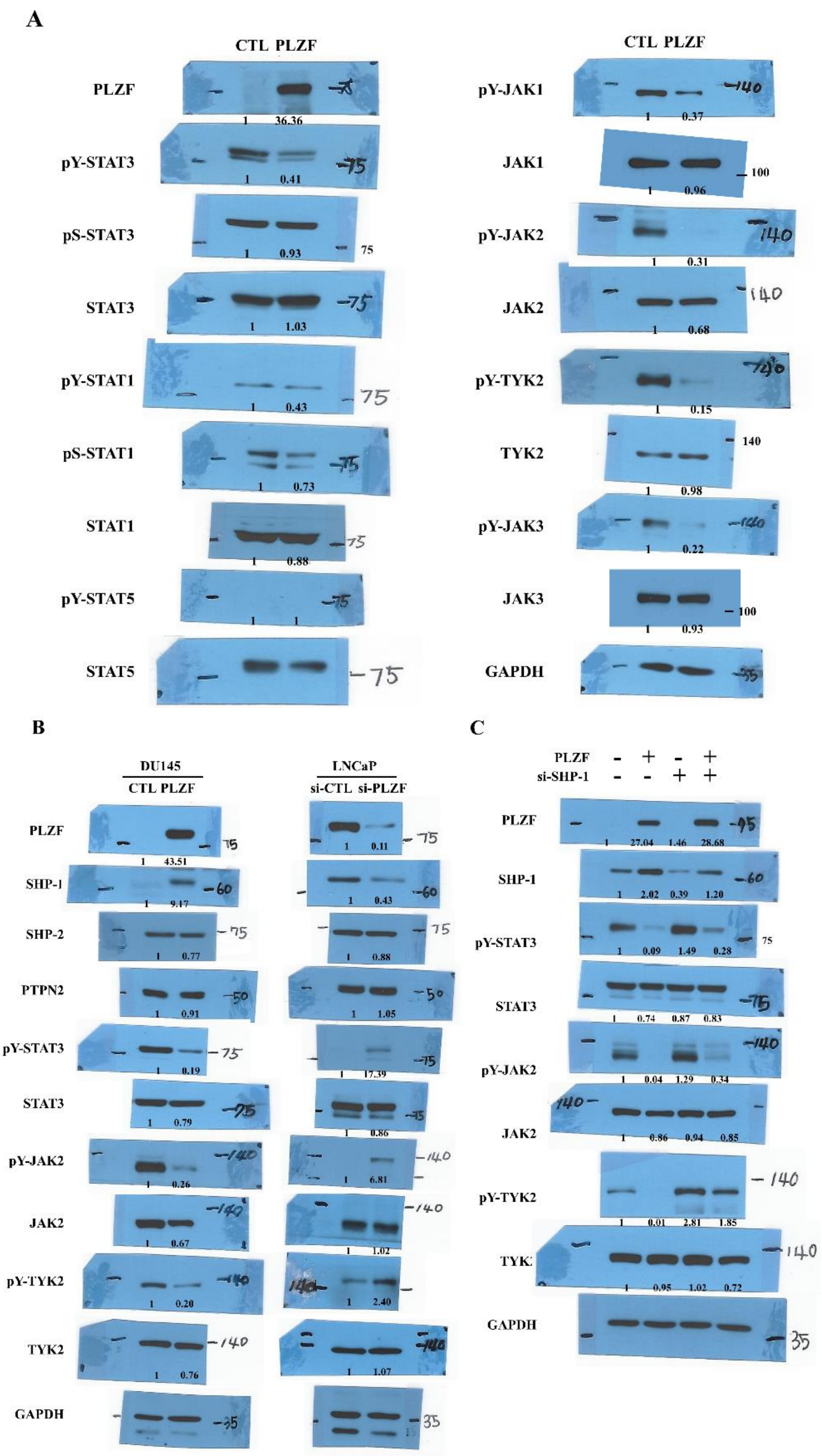

Figure S8. Original blots for western blot analyses shown in Figure 4: (A) Figure 4A, (B) Figure 4B, (C) Figure 4D.

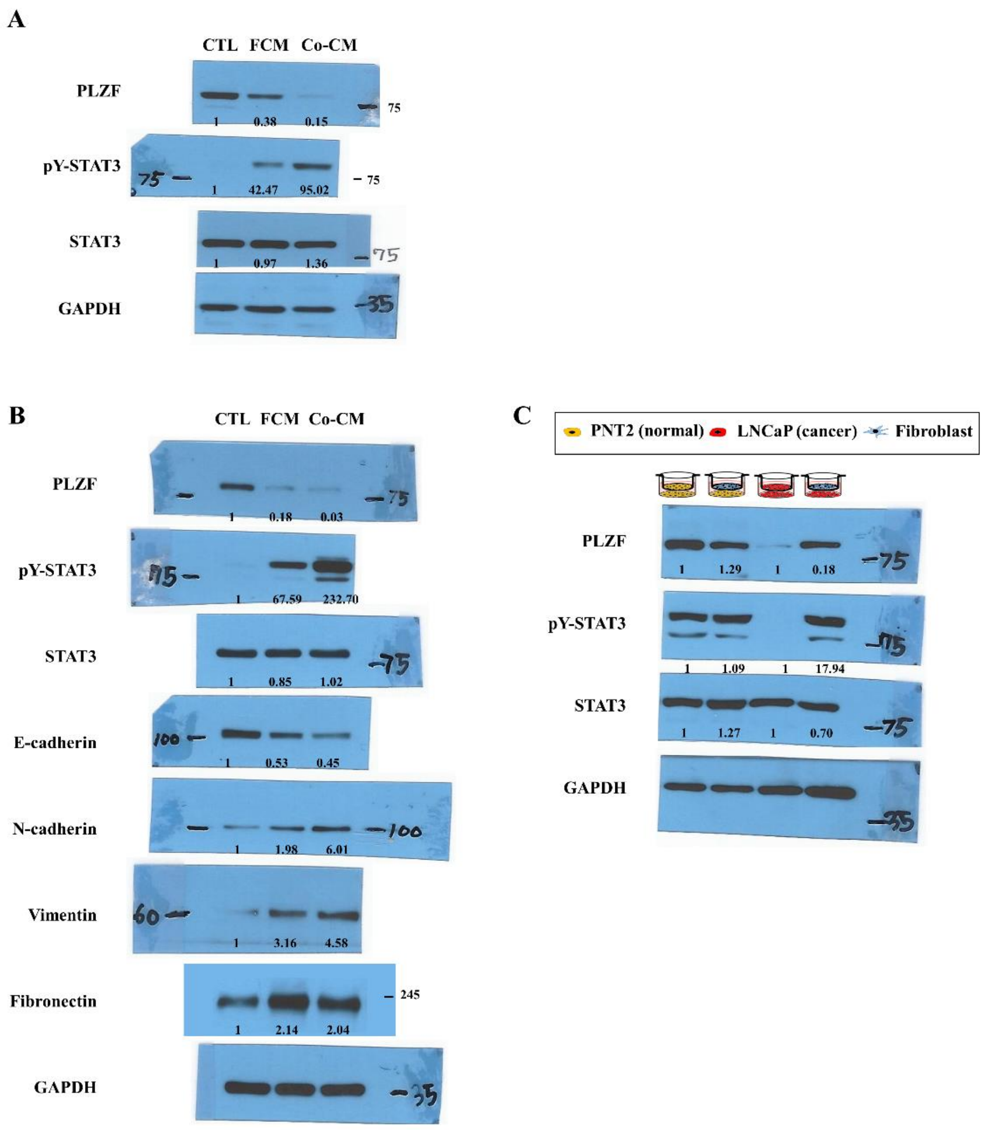

Figure S9. Original blots for western blot analyses shown in Figure 5: (A) Figure 5B, (B) Figure 5F, (C) Figure 5H.

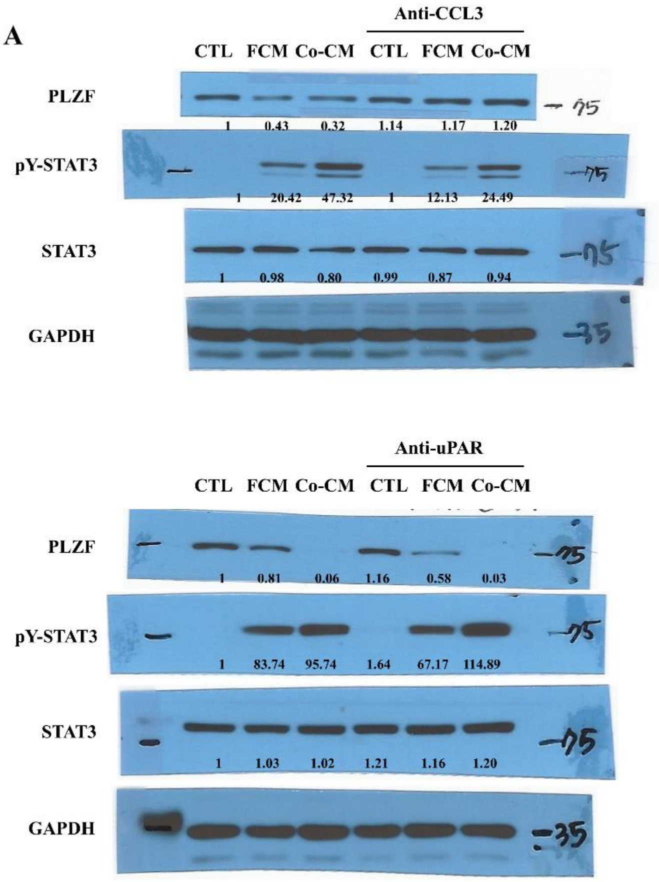

Figure S10. Original blots for western blot analyses shown in Figure 6: (A) Figure 6C.

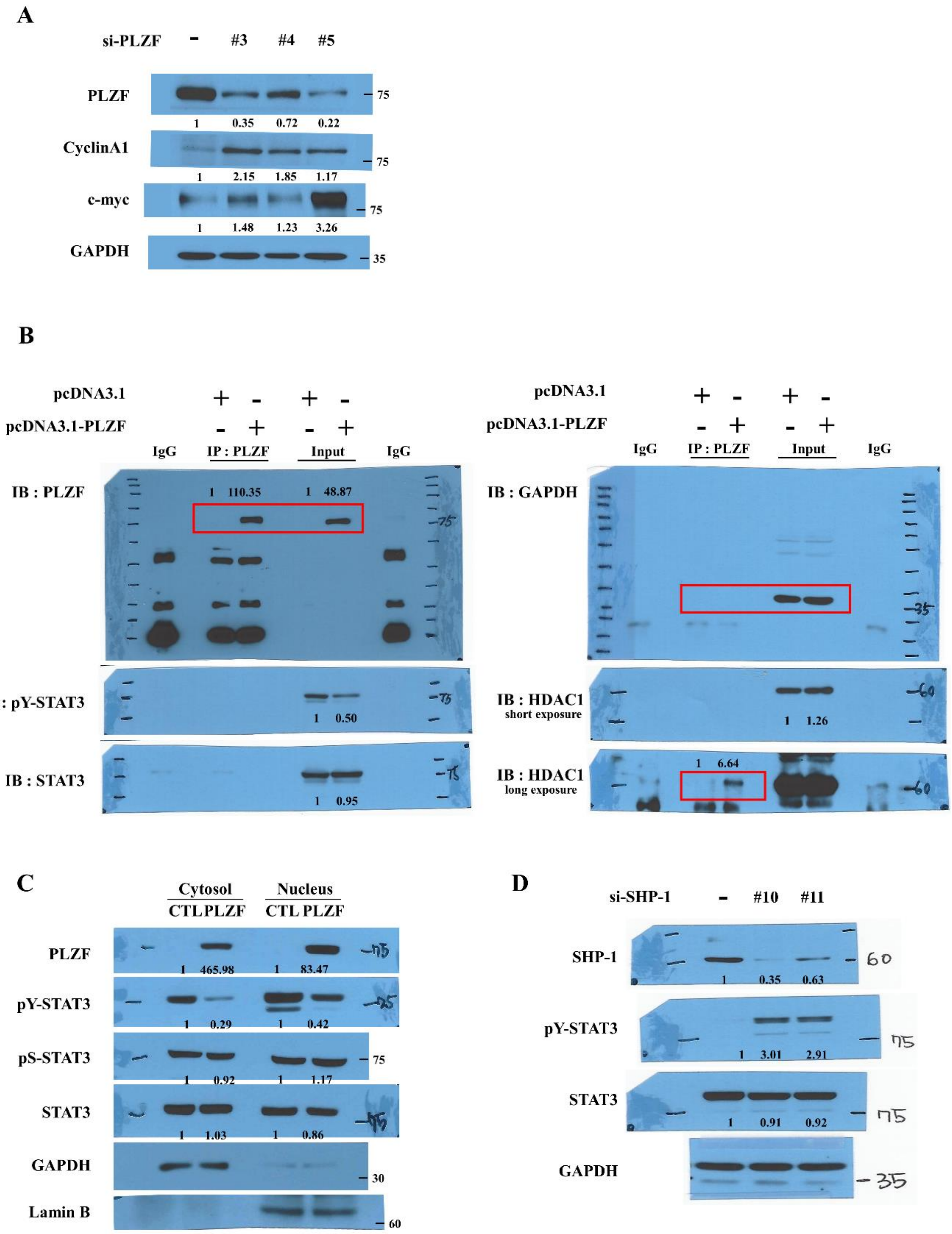

Figure S11. Original blots for western blot analyses shown in Figure S1 and S2: (A) Figure S1E, (B) Figure S2E, (C) Figure S2F, (D) Figure S2G.

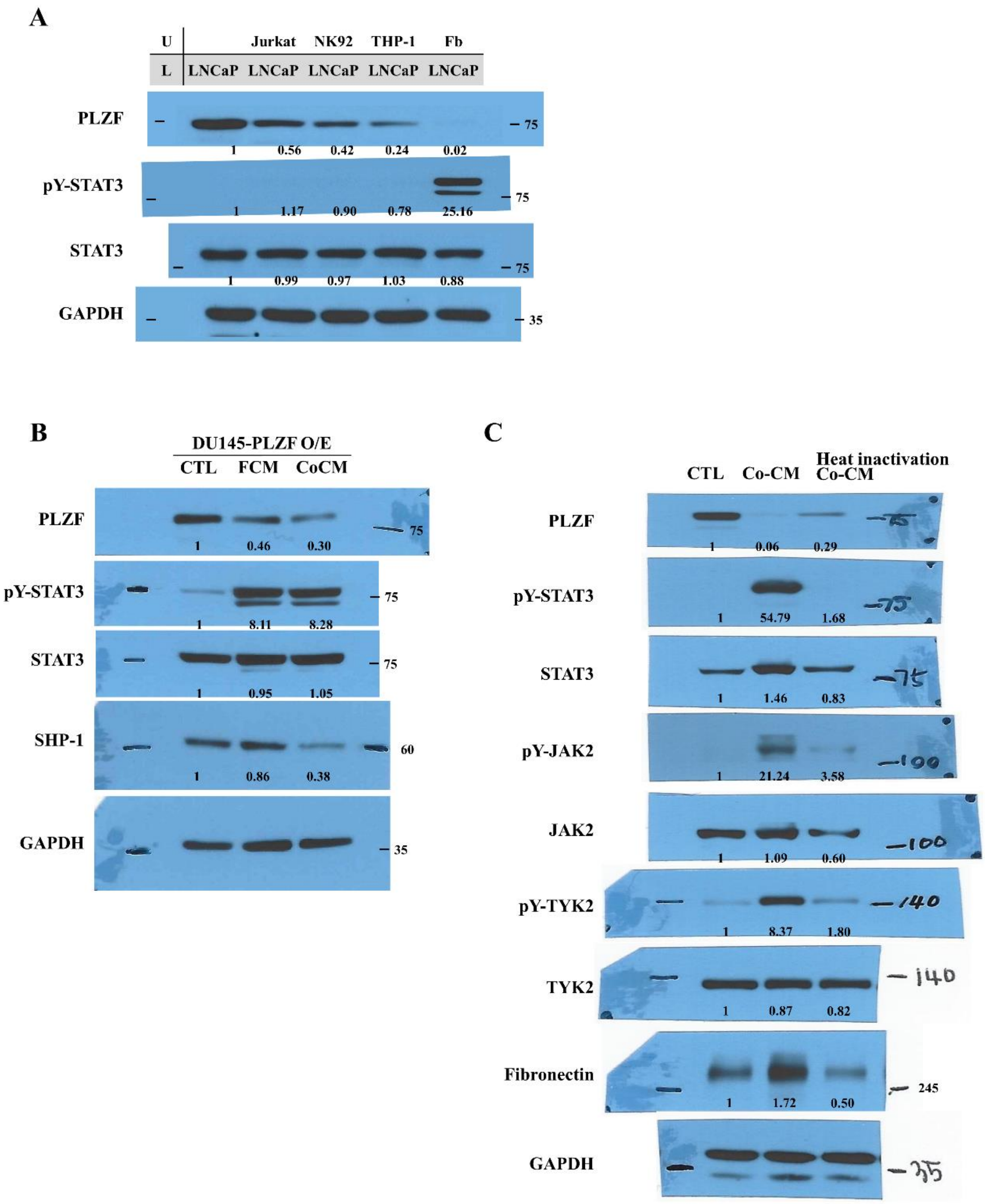

Figure S12. Original blots for western blot analyses shown in Figure S3: (A) Figure S3A, (B) Figure S3B, (C) Figure S3E.

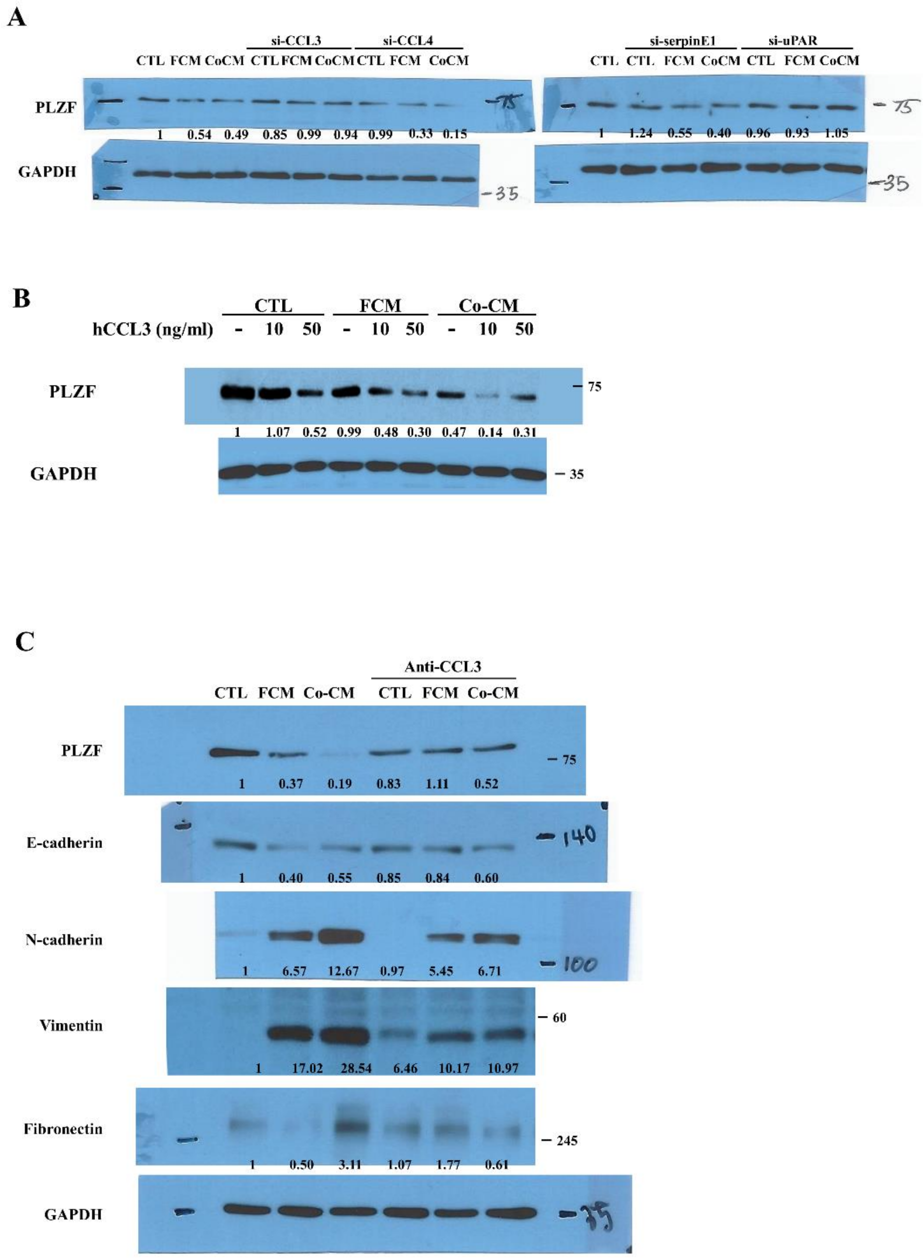

Figure S13. Original blots for western blot analyses shown in Figure S4: (A) Figure S4B, (B) Figure S4D, (C) Figure S4E.

3. Supplementary Table

Table S1. PSA levels information of prostate cancer patients.

No.	PSA (ng/ml)	Follow-up months	Follow-up result	Cause of death	Remark	Sex	Age
1	11.2	60	alive	.	.	M	60
2	30	60	alive	.	.	M	64
3	60.4	55	alive	.	.	M	71
4	7.4	47	alive	.	.	M	64
5	9.8	44	alive	.	.	M	59
6	34.9	43	alive	.	.	M	65
7	48.1	42	alive	.	.	M	73
8	10.6	42	alive	.	.	M	69
9	37.3	39	alive	.	.	M	62
10	1.2	39	alive	.	.	M	66

11	40	39	alive	.	.	M	60
12	8.4	39	alive	.	.	M	66
13	7	37	alive	.	.	M	70
14	17.5	23	dead	cancer	.	M	65
15	13.1	34	alive	.	.	M	67
16	1.1	33	alive	.	.	M	69
17	11.8	33	alive	.	.	M	63
18	17.6	27	alive	.	.	M	69
19	9	26	alive	.	.	M	70
20	5.8	26	alive	.	.	M	58
21	15.8	24	alive	.	.	M	58
22	31.4	24	alive	.	.	M	71
23	14.4	19	alive	.	.	M	70
24	18.3	18	alive	.	.	M	59
25	16.6	17	alive	.	.	M	63
26	.	16	alive	.	.	M	72
27	10.8	17	dead	cancer	.	M	66
28	10.8	16	alive	.	.	M	70
29	.	15	alive	.	.	M	70
30	26.9	15	alive	.	.	M	68
31	.	15	alive	.	.	M	63
32	25	15	alive	.	.	M	57
33	16.8	15	alive	.	.	M	72
34	0.5	15	alive	.	.	M	70
35	98	15	alive	.	.	M	75
36	.	15	alive	.	.	M	62
37	91	14	alive	.	.	M	63
38	161	17	dead	cancer	.	M	53
39	13	13	alive	.	.	M	63
40	.	11	alive	.	.	M	44
41	.	.	.	.	benign	M	69
42	.	.	.	.	benign	M	62
43	.	.	.	.	benign	M	66
44	.	.	.	.	benign	M	65
45	.	.	.	.	benign	M	69
46	.	.	.	.	benign	M	70
47	.	.	.	.	benign	M	70
48	.	.	.	.	benign	M	63
49	.	.	.	.	benign	M	44

© 2020 by the authors. Licensee MDPI, Basel, Switzerland. This article is an open access article distributed under the terms and conditions of the Creative Commons Attribution (CC BY) license (http://creativecommons.org/licenses/by/4.0/).
